# Aging of the Vascular System and Neural Diseases

**DOI:** 10.3389/fnagi.2020.557384

**Published:** 2020-09-29

**Authors:** Chisato Watanabe, Tsutomu Imaizumi, Hiromi Kawai, Kazuma Suda, Yoichi Honma, Masamitsu Ichihashi, Masatsugu Ema, Ken-ichi Mizutani

**Affiliations:** ^1^Laboratory of Stem Cell Biology, Graduate School of Pharmaceutical Sciences, Kobe Gakuin University, Kobe, Japan; ^2^Department of Stem Cells and Human Disease Models, Research Center for Animal Life Science, Shiga University of Medical Science, Shiga, Japan; ^3^Basic Research Development Division, Rohto Pharmaceutical Co., Ltd., Osaka, Japan; ^4^Institute for the Advanced Study of Human Biology (ASHBi), Kyoto University Institute for Advanced Study, Kyoto, Japan

**Keywords:** vascular system, capillary vessels, neocortex, vascular aging, neural diseases

## Abstract

Vertebrates have acquired complex high-order functions facilitated by the dispersion of vascular and neural networks to every corner of the body. Blood vessels deliver oxygen and nutrients to all cells and provide essential transport systems for removing waste products. For these functions, tissue vascularization must be spatiotemporally appropriate. Recent studies revealed that blood vessels create a tissue-specific niche, thus attracting attention as biologically active sites for tissue development. Each capillary network is critical for maintaining proper brain function because age-related and disease-related impairment of cognitive function is associated with the loss or diminishment of brain capillaries. This review article highlights how structural and functional alterations in the brain vessels may change with age and neurogenerative diseases. Capillaries are also responsible for filtering toxic byproducts, providing an appropriate vascular environment for neuronal function. Accumulation of amyloid β is a key event in Alzheimer’s disease pathogenesis. Recent studies have focused on associations reported between Alzheimer’s disease and vascular aging. Furthermore, the glymphatic system and meningeal lymphatic systems contribute to a functional unit for clearance of amyloid β from the brain from the central nervous system into the cervical lymph nodes. This review article will also focus on recent advances in stem cell therapies that aim at repopulation or regeneration of a degenerating vascular system for neural diseases.

## Introduction

The brain’s vascular system is highly organized to efficiently deliver oxygen and glucose to its tissues. Anatomically, dense vascular networks of arteries and veins are found in the pia mater, whereas the parenchyma contains only capillaries. The basic structure of the blood vessels in the neocortex is the delivery of blood from the cortical surface by the pial vessels into the parenchyma perpendicularly by the periventricular vessels and drainage to the surface. Generally, arteries that supply blood to the brain branch into smaller arterioles, which eventually branch into the smallest blood vessels, known as capillaries. Capillaries carry oxygen and nutrients to the surrounding neural cells, and the capillary network is where the majority of the molecular exchange occurs between blood and tissue. A recent study showed that capillary endothelial cells (ECs) in the aged brain exhibit transcriptional change primarily, in comparison with arterial and venous cells (Chen et al., 2020).

**Figure 1:**
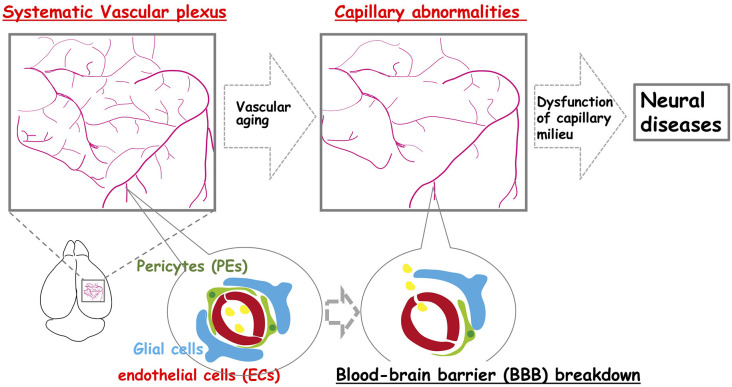
The neocortex-specific capillary structure is constructed during development but is broken down with aging. The vascular network is constructed to create a spatiotemporal capillary milieu in the brain. However, age-related structural decline in the integrity of the vascular system including vascular rarefaction contributes to various neural diseases.

**Figure 2:**
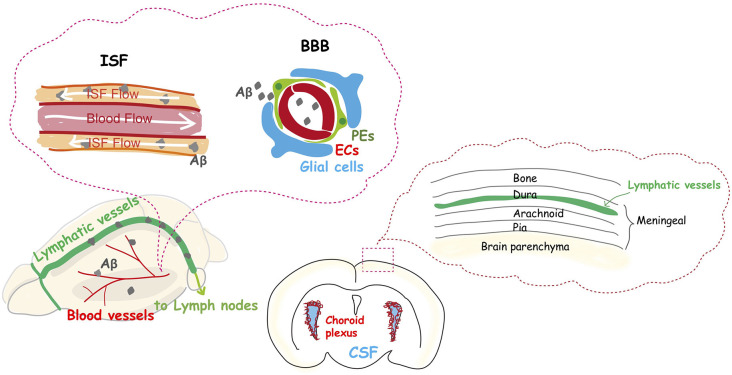
The dysfunction of the vascular system is an integral part of Alzheimer’s disease. The glymphatic system and meningeal lymphatic systems contribute to a functional unit for clearance of amyloid β from the brain into the cervical lymph nodes, and their function declines with aging. Aβ protein is deposited in and around capillaries, and its accumulation in the brain is largely considered the main cause of Alzheimer’s disease.

What happens to the brain when blood vessels are lost? More than one hundred years ago, Osler (1898) stated, “A man is as old as his arteries”. Tissues cannot survive without blood vessels to supply sufficient oxygen and nutrients. This is the case during embryonic development, for example, when the unfavorable distribution of vascular networks interferes with the normal development of organs. In contrast, brain vasculature undergoes many structural and functional alterations during aging (Figure 1). For example, capillary density declines (Klein and Michel, 1977; Wilkinson et al., 1981; Reeson et al., 2018), neovascularization potential attenuates (Frenkel-Denkberg et al., 1999; Rivard et al., 1999, 2000; Gao et al., 2009), plasma-derived circulatory cues become impaired (Villeda et al., 2011; Katsimpardi et al., 2014; Castellano et al., 2017), blood-brain barrier (BBB) permeability increases (Villeda et al., 2011; Lee P. et al., 2012), and the cerebral blood flow (CBF) decreases (Tarumi and Zhang, 2018). Also, recent studies suggest that structural and functional lymphatic vessels lining the dural sinuses drain macromolecules from the central nervous system (CNS) into the cervical lymph nodes (Aspelund et al., 2015; Louveau et al., 2015; Sun et al., 2018; Figure 2). Mixed pathologies of both Alzheimer’s disease (AD) and vascular abnormalities are the most common cause of clinical dementia in the elderly (Attems and Jellinger, 2014; Bennett et al., 2018; Sweeney et al., 2018a). Herein, we discuss these and also explore the recent advance of stem cell therapy that targets neovascularization during neural diseases.

## Systematic Vascular Plexus Is Constructed During Neocortical Development and Maintained to Exert Diverse Functions

The neocortex is one of the most sophisticated brain tissues. It consists of a horizontal six-layered structure, separated by cellular subtypes and neuronal projections (Bayer and Altman, 1991). The mammalian brain possesses characteristic regions of neural stem and progenitor cells (NSPCs), including the ventricular zone (VZ), and the subventricular zone (SVZ), which lines the lateral ventricles. NSPCs proliferate and self-renew to give rise to neurons, glia, and oligodendrocytes for normal brain development. An appropriate balance between self-renewal and differentiation is crucial for stem cell functions to generate precise cellular diversity in the neocortex (Mizutani et al., 2007; Franco and Müller, 2013; Inoue et al., 2017). The specific microenvironment where these stem cells are localized, the so-called stem cell niche, regulates quiescence, activation, differentiation, and cell fate. Recent studies have shown that one of the prominent components of the regulatory niche is the vascular niche (Vasudevan et al., 2008; Won et al., 2013; Ottone et al., 2014; Bjornsson et al., 2015; Tan et al., 2016), which creates specialized microenvironments *via* physically direct contact (Tavazoie et al., 2008; Komabayashi-Suzuki et al., 2019) and secreted-soluble factors (Shen et al., 2004, 2008; Kokovay et al., 2010).

Blood vessels have a relatively simple structure consisting of ECs that are surrounded by a basal lamina and an outer layer of pericytes (PEs). Blood vessel patterns vary markedly among different tissues and organs. The diversity and plasticity of vascular networks are considered important for this system to perform its distinct functions in each tissue and organ (Takashima et al., 2019). Evidence has shown that ECs, which show remarkable structural and functional heterogeneity, may be responsible for this diversity (Aird, 2007). Recent studies have demonstrated that molecular profiles define the heterogeneity of ECs in the capillaries found in different tissues (Nolan et al., 2013; Vanlandewijck et al., 2018). These heterogeneous differences were confirmed by both *in vitro* differentiation and an *in vivo* transplantation system (Nolan et al., 2013).

Oxygen level is a particularly important element in the regulation of ECs and PEs and determines how tissue vascularization is constructed. In hypoxic cells, hypoxia-inducible factor-1α (HIF-1α) is activated (Semenza and Wang, 1992) and drives vascular endothelial growth factor (VEGF) and other hypoxia-responsive genes. It also regulates the recruitment of endothelial progenitor cells in the endothelial lining of blood vessels to vascularization sites (Kelly et al., 2003). It has been suggested that these dynamic changes in expression during the developmental process play an important role in the construction of the brain’s vascular network (Gustafsson et al., 2005; Li et al., 2014; Wagenführ et al., 2015; Lange et al., 2016), but its specific details of this process have not yet been discovered.

CNS cells, including neurons and glial cells (e.g., astrocytes and microglia), closely interact with angiogenesis and vasculogenesis (Ma et al., 2013; Takahashi et al., 2015; Tan et al., 2016; Himmels et al., 2017). In a recent study, we found that an avascular region without a capillary invasion was specifically constructed in the VZ where mitotic progenitors are located, and NSPCs transiently expressed HIF-1α and VEGF, thereby attracting vascular endothelial tip cells (Komabayashi-Suzuki et al., 2019). The expression level of these proteins in the VZ gradually decreased, while their levels gradually increased in the intermediate zone (IZ) at later developmental stages (Komabayashi-Suzuki et al., 2019). Another recent study demonstrated that HIF-1α stabilization is required for the maturation arrest of oligodendrocyte progenitor cells (OPCs) through *Wnt7a/7b* activation (Yuen et al., 2014). Furthermore, we showed that OPCs come into contact with ECs frequently in the IZ and that the spatiotemporal HIF-1α activation corresponds with the timing of OPC maturation (Komabayashi-Suzuki et al., 2019). This suggests that the spatiotemporal regulation of HIF-1α and VEGF expression plays an essential role in the cytoarchitecture of both the vascular system and the neural system in the neocortex.

## The Brain-Specific Capillary Structure Breaks Down with Aging

The structural integrity of the vessels declines with age (Figure 1). Additionally, there is considerable evidence of declines in capillary density throughout the aged brain (Klein and Michel, 1977; Wilkinson et al., 1981; Reeson et al., 2018). Current evidence suggests that neocortical microvascular pathologies, such as age-related structural and functional declines in the vascular plexus (e.g., vascular rarefaction), contributes to age-related cognitive dysfunction and neurodegeneration (van Dijk et al., 2008; Brown and Thore, 2011). Moreover, there appears to be an age-related decline in the neovascularization capacity by various mechanisms. For example, HIF-1α becomes less responsive to hypoxia during aging. VEGF also becomes less responsive during aging, even in the presence of sufficient VEGF levels. This is probably due to an intrinsic change in VEGF-receptor 2 (Gao et al., 2009), and a reduction in the expression level of VEGF itself (Frenkel-Denkberg et al., 1999; Rivard et al., 2000). Hence, the potential for tissues to undergo vascular remodeling is age-related and results in decreased blood flow in the aged neocortex (Katsimpardi et al., 2014). The mesenchyme homeobox gene, MEOX2, encodes a family of homeodomain transcription factors expressed in the vascular system (Gorski and Walsh, 2003). MEOX2 has the potential to regulate the expression of many target genes, and can therefore modify the phenotype, and function of vascular cells. Deletion of Meox2 leads to the reduction of brain capillary density, which causes resting CBF to diminish, and the angiogenic response to hypoxia in the brain to be lost (Wu et al., 2005). In adult vasculature, Meox2 is predominantly expressed in ECs and modulates angiogenesis and vascular network construction. Meox2 that has gained or lost its function can also cause the vascular structure to be altered (Wu et al., 2005; Gohn et al., 2017). Also, Meox2 expression in vascular smooth muscle cells (VSMCs) is downregulated in response to serum, growth factors, and vascular injury (Gorski et al., 1993; Weir et al., 1995). These studies support a role for Meox2 in the maintenance of vascular integrity.

It is well known that age-related endothelial dysfunction is associated with prominent changes in the BBB, and aberrant activities within the neurovascular unit (NVU; Cai et al., 2017; Li et al., 2019). The BBB is a complex functional and anatomical structure composed of brain microvascular ECs that communicate with PEs and astrocytes, which enables these cells to organize into a well-structured NVU with neurons (Banks et al., 2018; Figure 1). Cerebral capillary ECs contain tight junctions, which tightly sealed cell-to-cell contact between adjacent ECs to form a continuous vascular system. This tight seals between cells lead to high endothelial electrical resistance, and low paracellular/transcellular permeability (Zlokovic, 2008). Recent studies have indicated that Wnt/β-catenin signaling is a key pathway required for both the formation of BBB functions, and the maintenance of BBB integrity (Engelhardt and Liebner, 2014; Liebner et al., 2018). The binding of the Wnt ligands, Wnt7a and Wnt7b, produced by neurons and astrocytes in the brain (Zhang et al., 2014) to the corresponding Wnt receptor, Fzd4, and Wnt co-receptor, LRP5/6, triggers the activation of Wnt/β-catenin signaling in BBB formation and function (Liebner et al., 2008; Stenman et al., 2008; Daneman et al., 2009). Also, GPI-anchored Reck and G protein-coupled receptor, Gpr124, have been implicated in Wnt7a/Wnt7b-specific signaling in mammalian CNS angiogenesis, BBB integrity, and function (Zhou and Nathans, 2014; Vanhollebeke et al., 2015; Tran et al., 2016; Chang et al., 2017; Cho et al., 2017; Vallon et al., 2018; Laksitorini et al., 2019).

## Vascular Dysfunction Is An Integral Part of Alzheimer’s Disease Pathogenesis

AD is the most common neurodegenerative disease, accounting for an estimated 60 to 80% of dementia cases. Emerging evidence indicates an important vascular contribution to AD, since Aβ protein is deposited in and around capillaries, and aberrant Aβ protein accumulation in the tissue is largely considered to be the main cause of AD (Liesz, 2019). Recent reports suggest that the common sporadic form of AD (late-onset), and some familial cases of AD (early onset) are characterized by elevated Aβ brain levels as a result of impaired Aβ elimination instead of overproduction (Mawuenyega et al., 2010). Normally, low Aβ levels in the brain are maintained through degradation, and elimination *via* its transvascular removal across the BBB, which results in the removal of 85% of Aβ. Removal of the remaining 15% normally occurs *via* the interstitial fluid (ISF) bulk flow along the outside of penetrating arteries, and subsequent cerebrospinal fluid (CSF) absorption in the circulatory and lymphatic system (Deane et al., 2004, 2008; Nelson et al., 2017; Figure 2).

Brain ECs with tight junctions form the BBB. It is generally believed that neurodegeneration is accompanied by BBB dysfunction (Figure 1), which begins as early as middle age in rodents and humans. Vascular damage is the initial insult, causing disrupted BBB function and impaired brain perfusion that contributes to neuronal injury, and dementia (Montagne et al., 2020; Musaeus et al., 2020). BBB disruption occurs as capillary leakage, the deregulation of ECs-PEs-glial communications, brain leukocyte infiltration, or aberrant angiogenesis (Armulik et al., 2011; Sweeney et al., 2018b). The cell-surface receptor, low-density lipoprotein receptor-related protein-1 (LRP1), has been reported to mediate Aβ endocytosis across the BBB (Kanekiyo et al., 2012, 2013), and Aβ transcytosis through the brain endothelium, as well as its subsequent systemic elimination *via* the liver, spleen, and kidney (Shibata et al., 2000). LRP1 expression in the brain and brain capillaries has been shown to decrease with age (Shibata et al., 2000; Deane et al., 2008; Silverberg et al., 2010; Storck et al., 2016), and its expression is further reduced in AD (Kang et al., 2000; Shibata et al., 2000). Aβ influx transport, from the plasma into the brain ISF occurs *via* the receptor for advanced glycation end products (RAGE), which promotes inflammation (Deane et al., 2003). Vascular aging in the brain of AD patients, which includes reduced capillary length, and decreased tight junction protein expression, might reflect aberrant angiogenesis potential that originated from the impairment of Meox2 expression in ECs (Zlokovic, 2011). In addition, BBB disruption upregulates transforming growth factor-α (TGF-α) signaling in astrocytes, resulting in neural dysfunction (Senatorov et al., 2019).

Interactions between ECs and mural cells, which include PEs and VSMCs, have recently come into focus as regulators of vascular formation, stabilization, remodeling, and function. Platelet-derived growth factor receptor-β (PDGFRβ) is predominantly expressed in the mural cells, such as capillary PEs and VSMCs. Expression levels of PDGFRβ in PEs are noticeably higher than those in VSMCs. Additionally, PEs control Aβ clearance from the brain. Their loss diminishes the removal of soluble Aβ and accelerates the onset and progression of disease pathogenesis in mouse models of AD (Sagare et al., 2013). PE-derived trophic supports that maintain a healthy brain might also be lacking in AD, and PE loss may contribute to a progressive, age-dependent, vascular-mediated neurodegeneration in animal models (Bell et al., 2010; Armulik et al., 2011; Nikolakopoulou et al., 2019). The numbers of PDGFRβ-positive PEs, PE coverage of the capillary, and the number of capillaries are all reduced in the AD patient brain, which shows evidence of a gene-dose effect associated with the number of APOE4 alleles (Sengillo et al., 2013). Brain capillary damage using a novel CSF biomarker of BBB-associated capillary PEs, PDGFRβ, and regional BBB permeability were developed in the hippocampus of individuals with early cognitive decline independent of Aβ and tau pathology, suggesting that BBB damage is an early biomarker of human cognitive dysfunction, including the early stages of AD (Nation et al., 2019). Furthermore, elevated PDGFRβ in the CSF was shown to predict cognitive decline in APOE4 carriers (Montagne et al., 2020).

The lymphatic drainage system was thought for many years to be absent in the mammalian brain. It is now accepted that meningeal lymphatic vessels remove macromolecules, such as Aβ protein, from the parenchyma into the cervical lymph nodes (Figure 2), due to the rediscovery and characterization of the CNS lymphatic system (Aspelund et al., 2015; Louveau et al., 2015). Furthermore, a recent study has shown that older mice have impaired brain perfusion of macromolecules compared with that of young mice, accompanied by a decrease in meningeal lymphatic vessel diameter and coverage (Da Mesquita et al., 2018). Another recent study demonstrated that basal meningeal lymphatic vessels are hotspots for the clearance of CSF macromolecules and that its function of drainage from the brain to the periphery is impaired with aging (Ahn et al., 2019). Moreover, the treatment of aged mice with VEGF-C enhanced drainage, which leads to learning and memory improvements (Da Mesquita et al., 2018). A study of the effects of the disruption of meningeal lymphatic vessels in AD mouse models confirmed that ablation led to the promotion of Aβ accumulation in the meninges (Da Mesquita et al., 2018), which is similar to human pathology.

## Cell Therapy Targeting of Neovascularization for Neural Disease Treatments

Collectively, these studies suggest that the structural and functional integrity of the vascular system is essential for normal brain function. Improvement of the vascular system may be a promising therapeutic strategy for improving neural disease treatment. There is now an enormous demand for new effective therapies in neural diseases, such as AD, because of a high and unmet medical need to treat these neural diseases. With this perspective, advances in stem cell-based therapies that aim to repopulate or regenerate a degenerating vascular system have been anticipated (Figure 1).

Mesenchymal stem cells (MSCs) have attracted attention due to their powerful intrinsic cell therapy properties, although the molecular mechanisms of their physiological action have not yet been clarified. Transplantation of MSCs derived from the human umbilical cord (Lee H. et al., 2012; Yang et al., 2013), placenta (Yun et al., 2013), and bone marrow (Naaldijk et al., 2017) has been reported to inhibit Aβ-induced cell death, reduce Aβ plaque size (Yang et al., 2013; Yun et al., 2013; Naaldijk et al., 2017), and rescue spatial learning and memory disorders (Lee H. et al., 2012; Yang et al., 2013; Yun et al., 2013) in rodent AD models. Another study demonstrated that intravenous administration of ischemia-tolerant MSCs displayed significant Aβ degradation, and had an anti-inflammatory impact in an AD mouse model (Harach et al., 2017). Intriguingly, both bone marrow-derived MSCs and adipose-derived stem cells (ASCs) have been shown to enhance vascular tube formation in a co-culture system (Ghajar et al., 2010; Verseijden et al., 2010; Duttenhoefer et al., 2013). Additionally, ASCs express angiogenic factors such as VEGF under hypoxic conditions (Rohringer et al., 2014). Moreover, MSCs have the potential to induce the differentiation of mural cells, such as PEs, by gap junction-dependent communication between MSCs and ECs (Hirschi et al., 2003). Also, bone marrow mononuclear cells (BM-MNC) have been shown to activate the repopulation or regeneration of neovascularization in ischemic tissue (Tateishi-Yuyama et al., 2002; Taguchi et al., 2003). Furthermore, a recent study has demonstrated that transplanted BM-MNCs activate angiogenesis through gap junction-mediated, direct cell-to-cell interactions between BM-MNC and ECs, followed by activation of HIF-1α, and suppression of autophagy in the ECs of ischemic brain tissue (Kikuchi-Taura et al., 2020). These studies suggest that stem cell-based therapies, which have utilized MSCs, ASCs, and BM-MNCs, have great potential for neovascularization in neural diseases.

In other words, it is possible to lead to the creation of new concepts on the development/progression of neural diseases, in which vascular aging is involved, by the effect of improving pathological conditions by the protective actions on neovascularization, including the response mechanisms, in which the brain capillaries undergo the actions by the physical contact between MSCs/ASCs/BM-MNCs and ECs, and by MSCs/ASCs/BM-MNCs-derived humoral factors.

## Conclusions and Perspectives

Interactions between vascular cells and neural cells play an essential role in both brain development and brain aging. In this review article, we summarized how ECs construct a vascular plexus, and that capillary networks with tissue-specific environments may control neocortical development, allowing each region to maintain various distinct functions. Capillaries in the neocortex are prone to obstruction with aging, and this event has a major impact on brain function. The dysfunction of the vascular system is an integral part of AD etiology and pathophysiology. Aβ has been thought to be mainly generated in the brain itself, but accumulating evidence suggests that Aβ is generated in both the brain and peripheral tissues (Bu et al., 2018). This underscores the relevance of the dysfunction of the vascular system, including lymphatic vessels, in AD.

In the future, to deeply understand brain aging/pathological conditions, it is necessary to elucidate further the progression mechanism of vascular aging by clarifying which linkage breakdown between the vascular system and neural system causes preferential degradation of any capillaries in any tissue. Also, by applying these basic findings, it is expected to lead to the establishment of new therapeutic concepts, such as stem cell therapy, and the development of the prevention of neural diseases/new therapeutics through the mechanism of inhibiting vascular aging.

## Author Contributions

All authors drafted the manuscript, discussed, and approved the final manuscript.

## Conflict of Interest

TI, HK, KS, and YH were employed by the Rohto Pharmaceutical Company Limited.

The remaining authors declare that the research was conducted in the absence of any commercial or financial relationships that could be construed as a potential conflict of interest.
